# Rheumatoid Synovial Fluids Regulate the Immunomodulatory Potential of Adipose-Derived Mesenchymal Stem Cells Through a TNF/NF-κB-Dependent Mechanism

**DOI:** 10.3389/fimmu.2019.01482

**Published:** 2019-06-28

**Authors:** Souraya Sayegh, Oula El Atat, Katy Diallo, Benjamin Rauwel, Yannick Degboé, Etienne Cavaignac, Arnaud Constantin, Alain Cantagrel, Viviane Trak-Smayra, Nada Alaaeddine, Jean-Luc Davignon

**Affiliations:** ^1^Centre de Physiopathologie de Toulouse Purpan, INSERM UMR 1043, Toulouse, France; ^2^Université Paul Sabatier Toulouse III, Toulouse, France; ^3^Faculté de Médecine, Université Saint-Joseph, Beirut, Lebanon; ^4^Centre de Rhumatologie, CHU de Toulouse, Toulouse, France; ^5^Centre de Chirurgie Orthopédique et Traumatologique, CHU de Toulouse, Toulouse, France; ^6^Faculty of Medical Sciences, Neuroscience Research Center, Lebanese University, Beirut, Lebanon

**Keywords:** mesenchymal stem cells, rheumatoid arthritis, synovial fluid, immunomodulation, TNF, NF-κB, Tregs, macrophages

## Abstract

**Introduction:** Adipose-derived mesenchymal stem cells (ADSC) have been shown to have remarkable immune-modulating effects. However, their efficacy in clinical trials has yet to be fully demonstrated. This could be due to a lack of a proper inflammatory environment *in vivo* that primes ADSC. Here, we define how the articular microenvironment of rheumatoid arthritis (RA) patients modulates the therapeutic efficiency of ADSC.

**Methods:** Synovial fluids (SF) were collected from 8 RA patients, 2 Spondyloarthritis patients and one control synovial fluid from a patient undergoing traumatic-related surgery. SF inflammatory status was determined by routine analysis and quantification of pro-inflammatory cytokines. ADSC were first treated with SF and ADSC proliferation and gene expression of immunomodulatory factors was evaluated. In order to determine the mechanisms underlying the effect of SF on ADSC, tumor necrosis factor (TNF), interleukin-6 (IL-6), and NF-κB neutralization assays were performed. To evaluate the effect of SF on ADSC functions, ADSC were pre-treated with SF and then co-cultured with either macrophages or T cells. The modulation of their phenotype was assessed by flow cytometry.

**Results:** Pro-inflammatory RASF maintained the proliferative capacity of ADSC and upregulated the gene expression of cyclooxygenase-2 (COX2), indoleamine-1,2-dioxygenase (IDO), interleukin-6 (IL-6), tumor-necrosis factor stimulated gene 6 (TSG6), intercellular adhesion molecule 1 (ICAM-1), vascular cell adhesion molecule 1 (VCAM-1), and programmed death-ligand 1 (PD-L1), all factors involved in ADSC immunomodulatory potential. The RASF-induced gene expression was mainly mediated by TNF alone or in combination with IL-6 and signaled through the NF-κB pathway. Conditioning ADSC with pro-inflammatory RASF enhanced their ability to induce CD4^+^Foxp3^+^CD25^high^ regulatory T cells (Tregs) and inhibit pro-inflammatory markers CD40 and CD80 in activated macrophages.

**Conclusions:** Inflammatory synovial fluids from RA patients had the capacity to modulate ADSC response, to induce Tregs and modulate the phenotype of macrophages. The clinical use of ADSC in affected joints should take into account the influence of the local articular environment on their potential. Having a sufficient pro-inflammatory microenvironment will determine whether optimal immunoregulatory response should be expected. Direct ADSC intra-articular delivery to patients could be a potential strategy to properly prime their immunomodulatory potential and enhance their clinical benefits.

## Introduction

Mesenchymal stem cells (MSC) are multipotent cells with the ability to differentiate into different cells of mesodermal lineage making them a potential candidate for regenerative therapy (1). Over the years, MSC have been successfully isolated from several tissues including bone marrow, umbilical cord, and adipose tissue (2, 3). Their undeniable appeal comes from the fact that they are immune-evasive with a lack of expression of MHC-II molecules (4), are easily expanded *ex vivo* (4) but most importantly, that they possess immune-modulating abilities through both the secretion of soluble mediators and cell-to-cell contact-dependent mechanisms (5).

MSC are not constitutively immunomodulatory but become so upon stimulation with pro-inflammatory cytokines, mainly interferon-γ (IFN-γ) alone or in combination with tumor necrosis factor (TNF) or interleukin-1β (IL-1β) (6, 7) or TNF alone (8). Following their activation, MSC suppress the proliferation and effector function of pro-inflammatory immune cells that orchestrate the pathophysiology of autoimmune diseases such as T lymphocytes, B lymphocytes, macrophages, dendritic cells, and natural killer cells (NK cells) (9–13). In fact, upon encountering a pro-inflammatory environment, they upregulate their production of chemokines and adhesion molecules such as vascular cell adhesion molecule 1 (VCAM-1) and intercellular adhesion molecule 1 (ICAM-1) (14). This leads to the recruitment and subsequent inhibition of pro-inflammatory cells through a co-inhibitory signal transmitted by programmed death-ligand 1 (PD-L1) for T cells (15) but also through MSC production of high levels of immune inhibitory factors such as prostaglandin E2 (PGE2), indoleamine-1,2-dioxygenase (IDO), interleukin-6 (IL-6), interleukin-10 (IL-10), transforming growth factor beta (TGF-β) and tumor-necrosis factor stimulated gene 6 (TSG6) for T cells, macrophages, and NK cells (5, 16, 17). Furthermore, MSC have been shown to not only suppress the proliferation of pro-inflammatory cells but also to generate regulatory T cells (Tregs) and skew macrophages to a pro-resolving anti-inflammatory profile as demonstrated *in vitro* in co-culture settings (18, 19) and *in vivo* in rheumatoid arthritis (RA) murine models such as collagen-induced arthritis (CIA) amongst others (20, 21).

Even though it has been well-established that MSC exert an immunomodulatory effect *in vitro* and in animal models of autoimmune diseases, this clear-cut effect is yet to be seen in human clinical trials (22, 23). This could be due to several factors, one of which is the lack of an appropriate inflammatory stimulus *in vivo* which hinders the proper activation of MSC (7). A proper delivery of MSC to inflamed sites could therefore help enhance their clinical application.

Rheumatoid arthritis (RA) is an autoimmune inflammatory disease characterized by chronic synovitis and progressive bone and cartilage destruction (24). The local inflammatory environment in rheumatoid joints is the result of an interplay between pro-inflammatory Th1 and Th17 cells, infiltrating macrophages that secrete pro-inflammatory cytokines and B cells production of autoantibodies (25). These clinical aspects are mirrored by a synovial fluid rich in pro-inflammatory mediators be it cytokines, growth factors and chemokines, immune complexes, damage-associated molecular patterns (DAMPs) or genetic material (microRNA) (26–28). All these mediators could have a potential role in directing the immunomodulatory function of MSC as either an inhibitor of inflammation or even a promoter when the absence of an adequate inflammatory environment leads to an insufficient production by MSC of anti-inflammatory factors.

Adipose-derived mesenchymal stem cells (ADSC) are MSC that share similar properties with bone marrow-derived stem cells (BM-MSC) but have the advantage of being more easily isolated with a higher cell yield and an increased proliferative capacity (29). As per our knowledge, there has been thus far no report on the behavior of ADSC within a synovial environment.

In this study, we addressed the role of the articular microenvironment of RA patients on ADSC efficiency as a therapeutic agent. We investigated whether synovial fluid from inflamed arthritic joints could modulate the proliferation and immunomodulatory potential of ADSC. Our findings demonstrate that having an ample pro-inflammatory synovial environment is of critical importance to enhance ADSC's therapeutic effects. This suggests that a direct intra-articular delivery to sufficiently inflamed joints would better contribute to the clinical use of ADSC in RA.

## Methods

### Synovial Fluid Collection

Synovial fluid was obtained from 8 RA patients, 2 Spondyloarthritis (SpA) patients and one control synovial fluid from a patient undergoing a traumatic-related surgery. Informed written consent was obtained and the study protocol was approved by the CHU Toulouse ethics committee (BioTOUL DC 2016-2804). RA patients fulfilled the criteria for the ACR/EULAR 2010 and SpA patients the criteria for the ASAS. All RA patients had active joint inflammation and were taken in charge in the Rheumatology Department of the Toulouse University Hospital. SF was collected during therapeutic arthrocentesis. Samples of SF were sent to the hospital laboratories for routine analysis. Analysis results along with patients' blood tests and medical history allowed a full assessment of inflammation taking place at the time of sample collection.

SF were transferred to heparin-treated tubes, treated with hyaluronidase 0.5 mg/mL (H3884, Sigma-Aldrich, St. Louis, MO, USA) for 10 min at room temperature and centrifuged to exclude cells and debris. Samples were then filtered on a pore size of 0.2 μm to exclude remaining macromolecules and frozen in aliquots at −80°C for later use.

### Adipose-Derived Mesenchymal Stem Cells Isolation and Characterization

Human ADSC were isolated from adult subcutaneous adipose tissue of patients undergoing elective abdominal lipectomy procedures as previously described (30). The study was carried out after obtaining informed written consent and the approval of the Université Saint-Joseph ethics committee (CEHDE 733). Briefly, adipose tissue samples were first digested in Hank's Balanced Salt Solution (Lonza, Walkersville, MD, USA) containing 0.075% collagenase type I (Sigma-Aldrich) for 1 h under gentle agitation at 37°C and then centrifuged to obtain the stromal-vascular fraction (SVF). SVF was filtered through a 100 μm nylon membrane, resuspended in Red blood cell lysis buffer (Sigma-Aldrich) for 10 min and washed with phosphate buffered saline (PBS, Lonza) and ethylenediaminetetraacetic acid (EDTA, Lonza). The isolated SVF were plated in T175 tissue culture flasks containing Dulbecco's modified eagle medium/nutrient mixture F-12 medium (DMEM/F12, Gibco, Invitrogen, Waltham, MA, USA) supplemented with 10% fetal bovine serum (FBS, Sigma-Aldrich) and 1% penicillin-streptomycin (PS, Lonza). Two days later, non-adherent cells were washed off and fresh media was added. Cells were trypsinized and plated for subculture when they reached 80% confluency. All experiments were performed using ADSC from early passages (P3–P5).

According to the criteria of the International Society for Cellular Therapy, ADSC were immunophenotyped for their cellular expression of CD73, CD105, CD90, CD44, CD45, CD31, CD34, and HLA-DR in addition to their multilineage differentiation capacity into chondrocytes, adipocytes and osteoblasts as previously described (30).

### Cytokine Quantification

Synovial fluid mediators were determined by screening pro-inflammatory cytokines on three SF using a membrane-based Human Cytokine Array kit (ARY005B, R&D Systems, Minneapolis, MN, USA). The chemiluminescent signals were visualized with a ChemiDoc XRS+ imaging system (Bio-Rad, Hercules, CA, USA) and analyzed using Image Lab 5.0. IL-6 and IFN-γ levels were quantified by ELISA according to the manufacturer's instructions (R&D Systems). Concentrations of MCP-1 levels were determined by Cytometric Bead Array (BD Biosciences, San Diego, CA, USA). Data was acquired on an LSRII cytometer (BD Biosciences) and analyzed using FCAP array v3 software (BD Biosciences). Quantification of IL-1β, IL-23, IL-12p70, IL-12p40, CCL17, CXCL10, IL-10, and IL-1RA in SF was performed by the LegendPlex Human M1/M2 Macrophage Panel (BioLegend, San Diego, CA, USA). Data was acquired on a MACSQUANT Q10 cytometer (Miltenyi Biotec, Bergisch Gladbach, Germany) and analyzed using Legendplex Data Analysis Software (BioLegend).

IL-6 and TIMP3 were quantified in ADSC culture supernatants by ELISA (R&D Systems).

### ADSC Proliferation Assay

Cell proliferation was assessed using a tetrazolium-based MTS colorimetric assay. In order to determine the best SF concentration to use, ADSC were first seeded in a 96 flat-bottomed well plate (10^4^ cells/well) for 24 h and then treated with serum-free media containing increasing concentrations of either SF control (CTL) or RA1 (5, 25, 50%) for 48 h. CellTiter 96 Aqueous One solution (Promega, Madison, WI, USA) was added for an additional 2 h and formazan crystals were quantified using the Varioskan Flash spectrophotometer (Thermo Fisher Scientific, Waltham, MA, USA). For all subsequent experiments, the different synovial fluids were used at a 5% concentration.

### ADSC and SF Treatment Protocols

To determine the effect of SF on the expression of immunomodulatory genes (Cyclooxygenase-2; COX2, IDO, IL-6, TSG6, TGF-B), immunosuppressive genes (ICAM-1, VCAM-1, PD-L1) or other genes expressed by ADSC (stanniocalcin1; STC-1, tissue inhibitor of metalloproteinases 3; TIMP3), ADSC were seeded in 24-well plates overnight. The following day, media was aspirated, and adherent cells were washed with PBS and cultured in serum-free media in the presence of 5% SF from either control, SpA, or RA patients for 24 h. For each experiment, SF control and SpA served as controls.

To determine the effect of TNF and IL-6 present in SF on ADSC gene expression, cells were cultured in the presence of SF CTL, RA1, or RA8 with or without anti-IL6R and/or anti-TNF (10 μg/mL) and RA8 in the presence of recombinant IL-6 (570 pg/mL) and TNF (50 pg/mL) (PeproTech, Rocky Hill, NJ, USA) added at the same concentrations that were detected in RA1. RA8 in the presence of recombinant TNF at 10 ng/mL served as a positive control. To assess the role of NF-kB signaling in the effect of SF on ADSC, cells were pre-treated with either vehicle or the specific inhibitor of NF-kB activation caffeic acid phenethyl ester CAPE 20 μM (Enzo Life Sciences, Farmingdale, NY, USA) for 2 h followed by 24 h stimulation with SF CTL, RA1, or RA8.

### Gene Expression Analysis

Total RNA was extracted from ADSC with High Pure RNA isolation kit (Roche Diagnostics GmbH, Mannheim, Germany) and 0.5 μg was reverse transcribed using RevertAid Minus Reverse Transcriptase (Thermo Fisher Scientific). Real-time quantitative PCR was performed with the Light Cycler 480 System Instrument using the LC480 SYBR green Master Mix (Roche Diagnostics GmbH). Primers were synthesized by Sigma Life Science (St. Quentin Fallavier, France) (Table S1). All values were normalized to the housekeeping gene RPS9 and expressed as fold change compared to SF control using the 2^−ΔΔCt^ method.

### Western Blot

ADSC were cultured for 6 h in the presence of SF CTL, RA1, or RA8. Protein extracts were lysed in 50 μL Laemmli Buffer, denatured at 95°C for 10 min and then sonicated. Extracts were run on Novex NuPAGE 4–12% Bis-Tris mini gels, transferred to a nitrocellulose membrane (Life technologies, Carlsbad, CA, USA) and probed with antibodies against IκB and Histone H3 (BioLegend) followed by HRP-conjugated secondary antibodies. The bands were visualized with a ChemiDoc XRS+ imaging system (Bio-Rad). All images were analyzed using Image Lab 5.0. IkB expression was normalized to expression levels of Histone H3 in each corresponding lane.

### Monocyte Isolation and Co-culture With ADSC

Monocytes were obtained from 6 healthy controls. All healthy controls were recruited from the Etablissement Français du Sang (Toulouse, France). Fresh peripheral blood mononuclear cells (PBMC) were isolated and CD14^+^ monocytes were purified using the MagniSort Human CD14+ positive selection kit (Life technologies). Monocyte purity was assessed by flow cytometry using an anti-CD14-FITC antibody and was routinely >90%. Cells were cultured in 24-well plates at (0.5 × 10^6^ cells/mL) and activated with LPS 20 ng/mL (PeproTech) and IFN-γ 25 ng/mL (Sigma-Aldrich) for 24 h.

To evaluate the effect of SF on the ability of ADSC to modulate macrophages, ADSC were plated in a 24-well plate and treated with either control SF or the different RASF for 24 h. Activated pro-inflammatory macrophages were washed and added the following day to ADSC treated with different conditions at a ADSC:macrophages ratio of 1:5 for another 24 h.

### T Cell Isolation and Co-culture With ADSC

CD4^+^ T cells were obtained from 7 healthy controls. PBMC were isolated by BD density gradient centrifugation and subsequently frozen in liquid nitrogen. CD4^+^ T cell were purified from frozen-thawed PBMC using the Magnisort Human CD4 T cell 2-step enrichment kit (Life Technologies) for positive magnetic separation. T cell purity was assessed by flow cytometry using an anti-CD4-FITC antibody (BD Biosciences). Cell purity was routinely >90%. T cells or PBMC were cultured in the presence or absence of ADSC at ADSC:T cells ratios of 1:40 and 1:5 and activated with beads coated with anti-CD3/CD28 for 72 h.

To evaluate the effect of SF on the ability of ADSC to modulate T cells, ADSC were plated in 96-well plates and stimulated for 24 h with SF control, RA1, or RA8 with or without anti-IL6R/anti-TNF or with the different RASF. The following day, adherent cells were washed with PBS and T cells were added at a ratio of 1:5 as previously described.

### Flow Cytometry Analysis

For intracellular cytokine detection, T cells were restimulated with PMA (50 ng/mL) and ionomycin (1 μg/mL, Sigma-Aldrich) in the presence of Brefeldin A (0.2%, BD Biosciences) for 4 h. Cells were then stained with antibodies against T cell markers or their respective isotype control anti-CD25-PECy7 and anti-CD4-FITC (BD Biosciences) for 20 min at 4°C in the dark, fixed, and permeabilized with the Transcription factor buffer set (BD Biosciences) and then stained intracellularly with anti-Foxp3-PE, anti-IFNγ-PE, and anti-IL17A-AF647 antibodies (BD Biosciences) for 30 min at 4°C for flow cytometry detection of Tregs, Th1, and Th17 cells, respectively.

For flow cytometric detection of macrophages, cells were stained with the following antibodies or their respective isotype controls anti-CD40-APCy7 (BioLegend), anti-CD80-BV421 (BioLegend), anti-CD16-V500 (BD Biosciences), anti-CD206-AF488 (BioLegend), anti-CD163-APC (Miltenyi Biotec) for 20 min at 4°C in the dark. All samples were acquired on a MACSQUANT Q10 cytometer (Miltenyi Biotec) and analyzed using FlowJo v7.6.5 software (Tree Star).

### Statistical Analysis

All results were expressed as mean ± SEM. After the Shapiro-Wilk normality test was performed, groups were compared using a Paired Student's *t*-test or Wilcoxon matched pairs test. To assess the correlation between concentrations of pro-inflammatory cytokines quantified in SF and ADSC gene expression, the data was square root-transformed (sqrt) to restore normal distribution and correlation was evaluated using the Pearson r correlation coefficient. RA4 TNF sqrt value was determined as an outlier and, taking into account the clinical particularity of this patient whose RA began as juvenile arthritis at a very young age (Table 1), it was excluded from correlation studies. Data were analyzed using GraphPad Prism v5.00 Software (GraphPad Software, San Diego, CA, USA). *P* values < 0.05 were considered as statistically significant.

**Table 1:** Characterization of SF based on patients' data.

	**Age range**	**Age range at diagnosis**	**Treatment**	**DAS28-CRP**	**SF nucleated cell count (/mm3)**	**RF**	**ACPA**	**Erosion**	**Other**
CTL									SF control from a patient undergoing non-inflammatory rheumatism-related surgery
SpA1	30–35	36-40	MTX		4,900			+	Spondyloarthritis
SpA2	66–70	Undetermined	Flurbiprofen		12,800				Psoriasis
RA1	60–65	50–55	Tocilizumab	5.77	15,200	+	+	+	
RA2	70–75	60–65	MTX, abatacept	6.58	8,250	+	+	+	
RA3	70–75	70–75	None	5.35	3,400	+	–	–	
RA4	26–30	0–5 (Juvenile arthritis)	MTX, etanercept	4.29	15,000	–	–	–	
RA5	76–80	40–45	MTX	3.84	4,000	+	–	+	
RA6	50–55	30–35	MTX, sulfasalazine, hydroxychloroquine	3.54	35,000	+	+	+	
RA7	80–85	60–65	None	4.26	9,500	+	+	+	
RA8	60–65	26–30	Prednisolone	3.89	19,400	+	–	–	

*SF were obtained from eight different RA patients, two SpA patients and one control SF from a non-rheumatologic joint. CTL, control; SpA, spondyloarthritis; RA, rheumatoid arthritis; DAS28-CRP, Disease Activity Score 28 using C-reactive protein; RF, rheumatoid factor; ACPA, anti-citrullinated protein antibodies; MTX, methotrexate*.

## Results

### Characterization of SF Based on Patients' Data and Level of Inflammation

**Figure 1:**
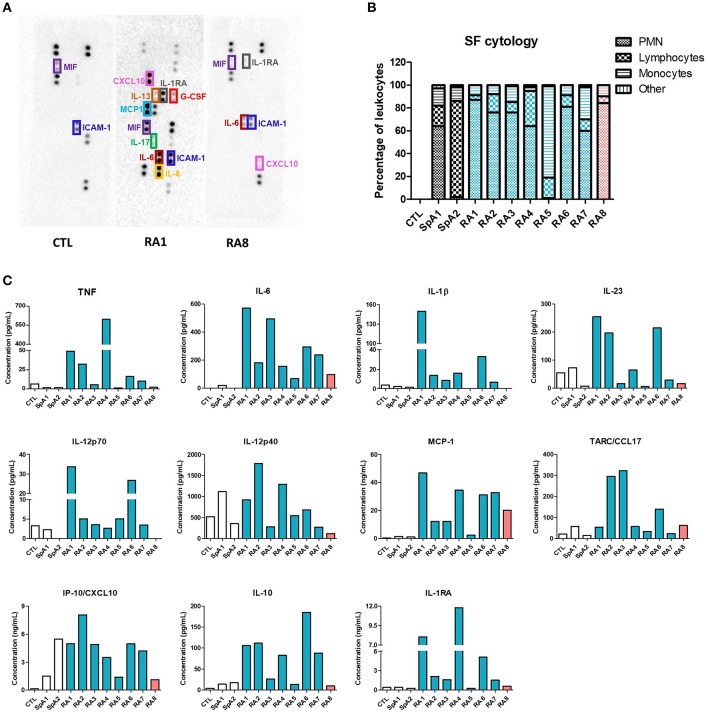
Evaluation of synovial fluids inflammatory status. SF were obtained from 8 different RA patients, 2 SpA patients and one control SF from a non-rheumatologic joint. RASF are listed depending on their inflammatory status (blue being very pro-inflammatory, orange slightly inflammatory). **(A)** Pro-inflammatory mediators were identified using an antibody-based membrane array in CTL, RA1, RA8 synovial fluids. Black spots on membrane represent each a cytokine and are semi-quantified in duplicates. Upper and lower rows represent reference spots. **(B)** SF cytology was determined after routine laboratory analysis. **(C)** TNF, IL-1β, IL-23, IL-12p70, IL-12p40, MCP-1, CCL17, CXCL10, IL-10, and IL-1RA were quantified in each SF by LegendPlex, MCP-1 was measured by Cytometric Bead Array and IL-6 by ELISA.

Clinical characteristics of patients and SF total nucleated cell counts are shown in Table 1. According to standard classification, SF is considered inflammatory when containing at least 2,000 cells/mm^3^ (26). As such, all SF in our study (with the exception of SF control) were considered as inflammatory. However, for our translational approach, the inflammatory status of SF was further characterized based on patients laboratory test results, Disease Activity Score using C-reactive protein (DAS28-CRP) values, and an initial screening of 36 different cytokines using a cytokine-profiling array (Figures 1A,B; Figure S1). Amongst the detected cytokines, TNF, IFN-γ, IL-1β, IL-23, IL-12p70, IL-12p40, CCL17, CXCL10, IL-10, and IL-1RA were all quantified by LegendPlex Array, MCP-1 was measured by CBA and IL-6 by ELISA. RA1–7 were confirmed as highly pro-inflammatory synovial fluids with high levels of quantified pro-inflammatory cytokines. RA1 presented the most inflammatory profile with considerably higher concentrations of TNF, IL-6, IL-1β, IL-23, IL12p70, and MCP-1. RA8 was determined as the least inflammatory RASF as it contained extremely low levels of TNF, IL-1β, IL-23p70 combined (Figure 1C). SF control was considered as a low-grade inflammatory negative control and SpA1, SpA2 as non-rheumatoid inflammatory controls with very low concentrations of TNF, IL-6, IL-1β, and MCP-1 detected in synovial fluids (Figure 1C). IFN-γ levels were undetected in all SF samples, as reported (31) which leads to believe that its role might not be prominent in the articular microenvironment and was thus not considered for the rest of the study (data not shown) (31).

### ADSC Phenotype Is Not Altered by SF

ADSC were able to differentiate into osteoblasts, adipocytes, and chondrocytes and were shown to be positive for CD90, CD105, CD73, CD29 and negative for CD44, CD45, CD34, and HLA-DR (Figure S2). In order to evaluate whether the phenotype of ADSC could be modulated by the different SF, ADSC were cultured in the presence of 5% SF for 24 h and the cellular expression of ADSC markers was assessed by flow cytometry. None of the SF treatments affected the expression of CD90, CD105, CD73, CD44, CD45, CD34, and HLA-DR suggesting that ADSC immune-evasive phenotype is not altered by SF (Figure S3).

### SF Differentially Affect ADSC Proliferation

**Figure 2:**
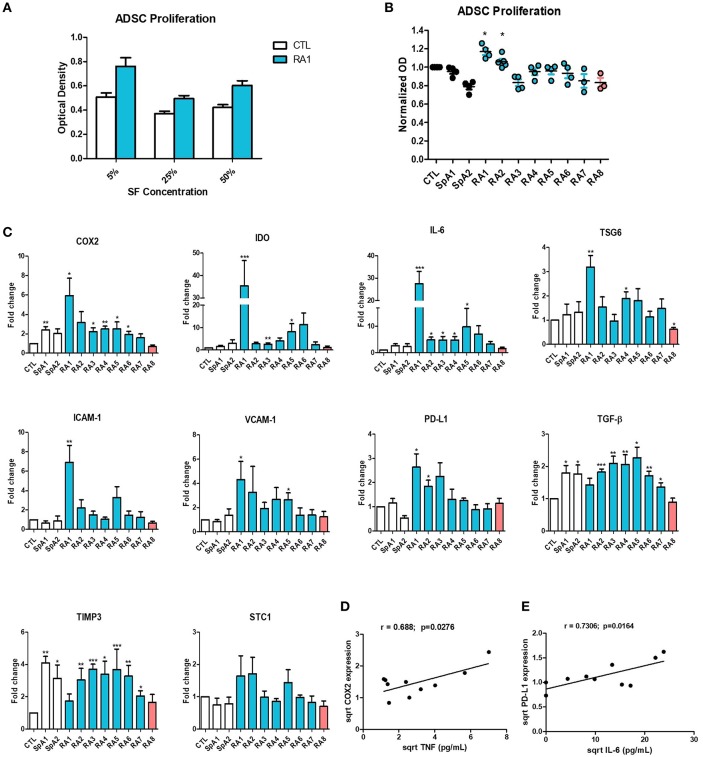
Differential effect of synovial fluids on ADSC proliferation and gene expression. **(A)** ADSC were cultured for 48 h in the presence of increasing concentrations of SF Control and RA1 at 5, 25, and 50% (*n* = 3). **(B)** For the remaining experiments, ADSC were cultured in the presence of 5% SF for 48 h from either RA, SpA, or control patients (*n* = 3 − 6). ADSC proliferation was assessed using a colorimetric MTT proliferation assay. **(C)** ADSC were cultured for 24 h in the presence of SF from either RA, SpA, or control patients. COX2, IDO, IL-6, TSG6, ICAM-1, VCAM-1, PD-L1, TGF-B, TIMP3, and STC-1 gene expression was evaluated by quantitative RT-PCR. RNA levels were normalized to RPS9 (*n* = 5). Results are represented as mean ± SEM. ^*^p < 0.05; ^**^p < 0.01; ^***^p < 0.001. **(D,E)** Correlation (Pearson) between **(D)** TNF concentrations and COX-2 gene expression and **(E)** IL-6 concentrations and PD-L1 gene expression. Data are square root-transformed.

We next studied the effect of SF on ADSC proliferation. First, we determined the optimal concentration to use by culturing ADSC in the presence of 5, 25, or 50% SF for 48 h. All concentrations had a similar effect on ADSC proliferation (Figure 2A) thus a 5% concentration was used for all subsequent experiments. SF maintained ADSC's proliferative properties. Only 2 pro-inflammatory RASF RA1 and RA2 slightly but significantly induced ADSC proliferation whilst the remaining had no inhibitory effect (Figure 2B).

### SF Differentially Affect ADSC Gene Expression

To assess the effect of SF on the expression of genes implicated in the immunomodulatory and immune-suppressive ability of ADSC, cells were cultured in the presence of either control, SpA, or RASF for 24 h. SpA SF slightly induced the expression of only COX2 and TGF-β compared to SF control (Figure 2C). On the other hand, pro-inflammatory RASF all significantly induced the expression of genes implicated in ADSC potential albeit to different extents. RA1, the most pro-inflammatory RASF, enhanced very potently the expression of COX2, IDO, IL-6, TSG-6, ICAM-1, VCAM-1, PD-L1, and TGF- β compared to control (up to 35-fold change) whereas RA2–RA7 induced more modestly their gene expression (up to 11-fold change). However, RA8 which was considered as the least inflammatory statistically inhibited the expression of TSG-6 and had a trend toward the inhibition of COX2, IDO, IL-6, ICAM-1, VCAM-1, PD-L1, and TGF-β. Other genes not directly implicated in ADSC immunomodulatory potential such as TIMP-3 and STC-1 were not differentially modulated by the different SF and were used as controls for the rest of the study (Figure 2C). SF effect on IL-6 and TIMP3 gene expression was confirmed by their quantification in ADSC culture supernatants (Figure S4). The enhanced expression of immunomodulatory genes by ADSC thus seems to be dependent on the pro-inflammatory profile of RASF. This was further confirmed by a significant correlation between TNF concentrations and COX-2 (Figure 2D), TSG6, VCAM-1, PD-L1 gene expression (Figure S5) and between IL-6 concentrations and PD-L1 expression (Figure 2E). We had a trend toward correlation with the rest of the aforementioned genes though not statistically significant due to the low number of samples (Figure S5).

### RASF-Induced Gene Expression in ADSC Is Mediated by TNF

**Figure 3:**
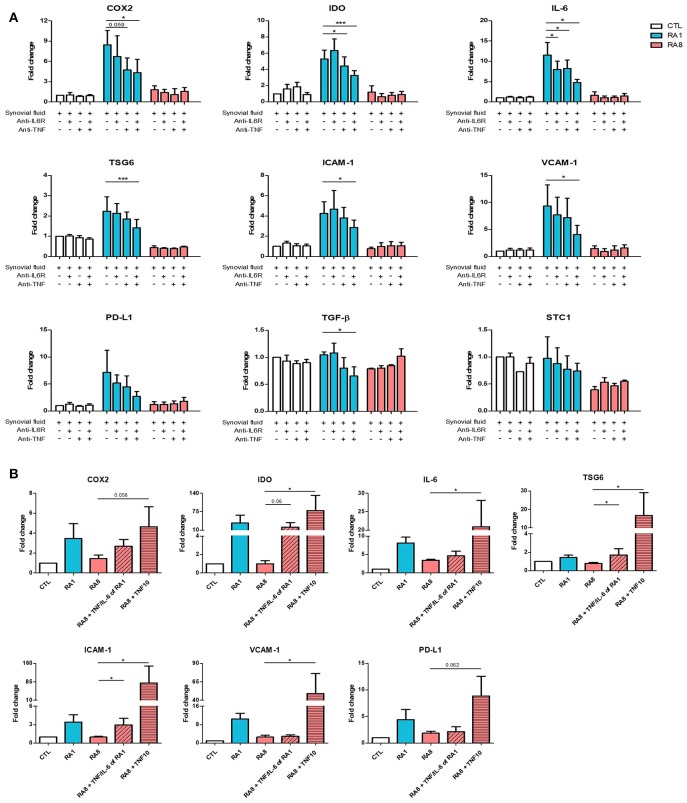
TNF mediates the enhancing effect of pro-inflammatory RASF on ADSC gene expression. ADSC were cultured for 24 h in the presence of SF CTL, RA1, or RA8 with or without anti-IL6R and/or anti-TNF **(A)** and SF CTL, RA1, and RA8 alone or in the presence of IL-6 (570 pg/ml) and TNF (50 pg/ml) as found in RA1 or TNF (10 ng/ml) **(B)**. COX2, IDO, IL-6, TSG6, ICAM-1, VCAM-1, PD-L1, TGF-B, TIMP3, and STC-1 gene expression was evaluated by quantitative RT-PCR. RNA levels were normalized to RPS9. Results are represented as mean ± SEM of at least 4 independent experiments. ^*^p < 0.05; ^***^p < 0.001.

Having previously detected TNF and IL-6 in RASF (Figure 1C), we investigated whether blocking these cytokines with neutralizing antibodies alone or in combination could inhibit RASF-induced gene expression in ADSC. To perform these experiments, three different SF were selected: SF control, RA1 as a pro-inflammatory inducer of ADSC immunomodulatory properties and RA8 as a low-inflammatory inhibitor. ADSC were cultured for 24 h in the presence of SF with or without anti-IL6R and/or anti-TNF. TNF and IL-6 neutralization alone in RA1 inhibited the induction of gene expression although it was not statistically significant due to donor variability (Figure 3A). A simultaneous neutralization of TNF and IL-6 in RA1 but not in control or RA8 resulted in an additive and significant inhibitory effect on ADSC gene expression (Figure 3A). We thus speculated that the presence of TNF and IL-6 in SF could be pivotal for the induction of ADSC potential. To further clarify the role of IL-6 and TNF, ADSC were treated with RA8 alone or in the presence of recombinant IL-6 (570 pg/mL) and TNF (50 pg/mL) as found in RA1 or in the presence of recombinant TNF at a concentration of 10 ng/mL. Mirroring data on inhibition, adding recombinant TNF (10 ng/mL) to RA8 as a positive control significantly induced the expression of IDO, IL-6, TSG-6, ICAM-1, VCAM-1. COX2 and PD-L1 gene expression was also induced although not significantly. On the other hand, adding IL-6 and TNF to RA8 at the same concentrations detected in RA1 partly overcame its suppressive effect on ADSC as shown by the induction of COX2, IDO, TSG6, and ICAM-1 (Figure 3B).

### RASF Modulates NF-κB Signaling Pathway in ADSC

**Figure 4:**
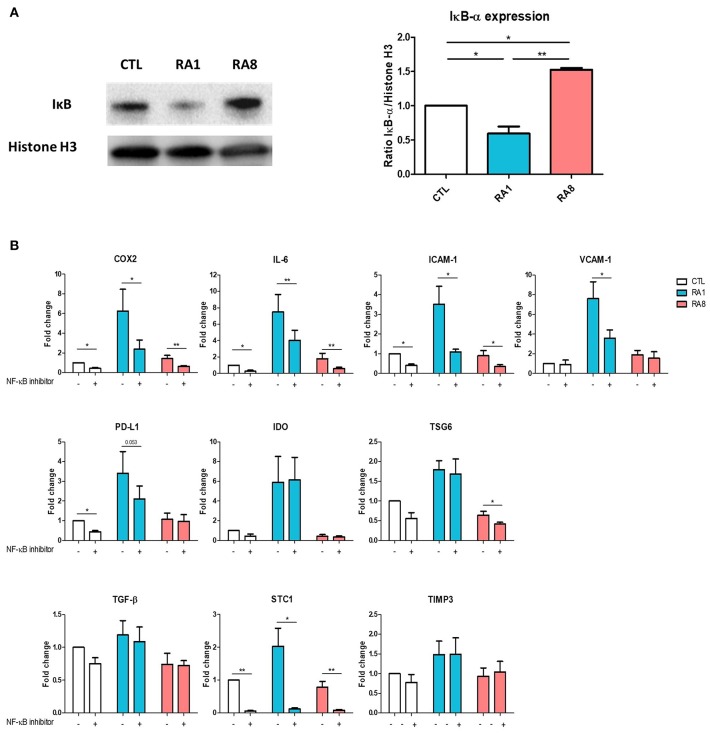
The effect of pro-inflammatory RASF partly signals through NF-κB. **(A)** ADSC were cultured for 6 h in the presence of SF CTL, RA1, or RA8. IκB expression was assessed by western blot. Histone H3 was used as control. **(B)** ADSC were pretreated with either vehicle or caffeic acid phenethyl ester CAPE (20 μM) for 2 h and then cultured for 24 h in the presence of SF CTL, RA1 or RA8. COX2, IDO, IL-6, TSG6, ICAM-1, VCAM-1, PD-L1, TGF-b, TIMP3, and STC-1 gene expression was evaluated by quantitative RT-PCR. RNA levels were normalized to RPS9. Results are represented as mean ± SEM of 3 independent experiments. ^*^p < 0.05; ^**^p < 0.01.

Because TNF plays an important role in activating the NF-κB signaling pathway, we first examined whether stimulating ADSC with the different SF could induce the expression of IκB which is inversely correlated with NF-κB activation. ADSC were treated for 6 h with SF control, RA1 and RA8. The expression of IκB was significantly inhibited in ADSC stimulated with RA1 compared to SF control suggesting an activation of NF-κB pathway. Conversely, RA8 significantly induced the expression of IκB indicating an inhibition of NF-κB (Figure 4A). To confirm the involvement of NF-κB in RA1-induced gene expression, we pre-treated the cells with the inhibitor of NF-κB activation CAPE prior to SF stimulation. We showed that by specifically inhibiting NF-κB activation, the effect of RA1 but not control or RA8 was statistically reversed. The induction of COX2, IL-6, ICAM-1, VCAM-1, PD-L1, and STC1 by RA1 was inhibited whereas IDO, TSG6, TGF-β, and TIMP3 gene transcripts remained unchanged (Figure 4B).

### Conditioning ADSC With RASF Influences Their Capacity to Inhibit Pro-inflammatory Markers in Macrophages

**Figure 5:**
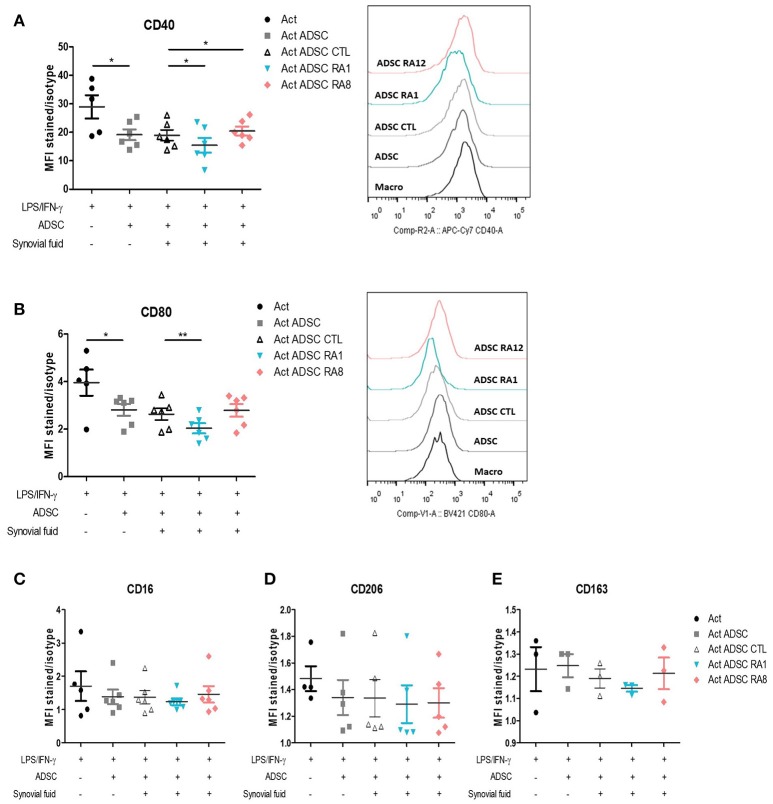
Conditioning ADSC with pro-inflammatory RASF enhances their ability to inhibit pro-inflammatory markers in macrophages. **(A–E)** ADSC were plated in 48-well plates and stimulated for 24 h with SF control, RA1, or RA12. The next day, macrophages from healthy donors were added to ADSC for 24 h following a 24-h activation with LPS/IFN-γ. Cells were then harvested and stained with anti-CD40 **(A)** and anti-CD80 **(B)** antibodies for flow cytometry detection of pro-inflammatory markers and anti-CD16 **(C)**, anti-CD206 **(D)**, anti-CD163 **(E)** for flow cytometry detection of alternative markers. Results are represented as mean ± SEM of 3–6 independent experiments. ^*^p < 0.05; ^**^p < 0.01.

We first validated the effect of ADSC on the modulation of macrophages pro-inflammatory markers. To that aim, we chose to study the cellular expression of CD40 and CD80 as pro-inflammatory markers, as described (32). Monocytes were first activated with LPS and IFN-γ for 24 h to skew them toward a pro-inflammatory phenotype and then added to ADSC at an ADSC:macrophages ratio of 1:5. In the co-culture setting, ADSC inhibited the expression of pro-inflammatory markers CD40 and CD80 in macrophages (Figures 5A,B). We thus evaluated whether stimulating ADSC with SF control, RA1, or RA8 for 24 h could affect their capacity to modulate these markers. ADSC conditioned with RA1 inhibited more potently the expression of CD40 and CD80 whereas ADSC conditioned with RA8 were less effective (Figures 5A,B). Similarly, all ADSC conditioned with RA2–RA7 significantly inhibited CD40 expression compared to control and to a lesser extent CD80 with RA1 being the most efficient in accordance with its superior effect on ADSC gene expression in Figure 2 and Figures S6A,B. These results indicate that RASF affects the capacity of ADSC to modulate pro-inflammatory macrophage markers. In parallel, ADSC did not affect the expression of alternative markers CD16, CD206, and CD164 (Figures 5C–E).

### Conditioning ADSC With RASF Affects Their Ability to Induce Tregs

**Figure 6:**
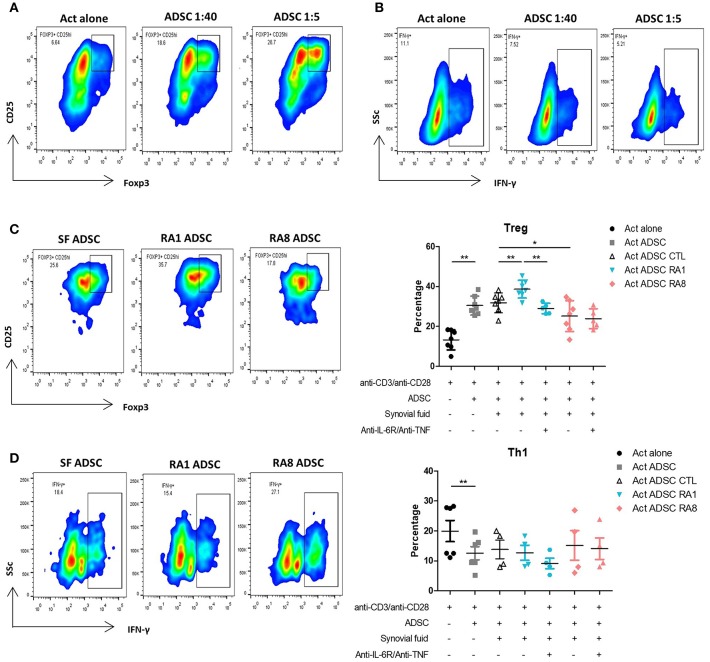
Conditioning ADSC with pro-inflammatory RASF enhances their ability to induce Tregs but not inhibit Th1. T cells from healthy donors were cultured in the presence or absence of ADSC at 1:40 and 1:5 ratios and activated with beads coated with anti-CD3/CD28 for 72 h. Cells were then harvested and stained with anti-CD25 and anti-Foxp3 antibodies for flow cytometry detection of Tregs (representative of 7 independent experiments) **(A)** or anti-IFNγ antibodies for flow cytometry detection of Th1 (representative of 6 independent experiments) **(B)**. **(C,D)** ADSC were plated in 96-well plates and stimulated for 24 h with SF control, RA1, or RA12 with or without anti-IL6R and anti-TNF. The following day, ADSC were washed and T cells were added to ADSC at a ratio of 1:5 and activated with beads coated with anti-CD3/CD28 for 72 h. Cells were harvested and Tregs **(C)** or Th1 **(D)** percentages were detected by flow cytometry. Results are represented as mean ± SEM of 4–7 independent experiments. ^*^p < 0.05; ^**^p < 0.01.

To examine the immunomodulatory effect of ADSC on different T cell subsets, purified CD4+ T cells were activated with beads coated with anti-CD3/CD28 at ADSC:T cells ratios of 1:40 and 1:5 for 3 days. At both ratios, ADSC significantly increased the percentage of Tregs and inhibited Th1 cells as detected by the expression of CD4, CD25^high^ Foxp3+ cells and IFN-γ+ cells, respectively (Figures 6A,B). ADSC did not have a particular effect on IL-17 producing cells (data not shown). These results were in accordance with an increase in T cell viability in the presence of ADSC (data not shown). We then investigated whether SF could functionally affect the ability of ADSC to modulate T cell subsets. ADSC were stimulated for 24 h with SF control, RA1, or RA8 with or without anti-IL6R and anti-TNF prior to the addition of T cells at a 1:5 ratio. As observed in the effect of SF on ADSC gene expression, ADSC conditioned with RA1 induced more potently Tregs compared to SF CTL whereas ADSC conditioned with RA8 were significantly less effective in the induction of Tregs. Furthermore, the enhancing effect of RA1 was abrogated in the presence of the neutralizing antibodies (Figure 6C). These results were confirmed in co-culture experiments with PBMC as well (Figure S7). In addition to RA1, ADSC conditioned with pro-inflammatory RA2 through RA7 were more efficient in inducing Tregs compared to control (Figure S8). This suggests that RASF differentially modulate the ability of ADSC to induce Tregs and that this effect is directly correlated to the presence of IL-6 and TNF in SF. We next assessed whether RASF could influence the effect of ADSC on Th1 cells. ADSC conditioned with RA1 did not enhance their ability to inhibit Th1 cells. However, ADSC conditioned with RA8 inhibited Th1 cells to a lesser extent though not statistically significant (Figure 6D).

## Discussion

In this study, we demonstrate that the immunomodulatory efficiency of ADSC is highly dependent on the cytokine/articular microenvironment in which they are present. Pro-inflammatory synovial fluids maintain the proliferation of ADSC and upregulate the expression of genes involved in their immunomodulatory potential through an TNF/NF-κB dependent mechanism. Furthermore, this study shows that ADSC exposed to a pro-inflammatory SF are more effective in inducing regulatory T cells and inhibiting pro-inflammatory macrophages compared to control SF.

Although ADSC are considered immune-evasive, it has been reported that they could upregulate their expression of MHC-II when treated with IFN-γ (33). Here, we show that none of the ADSC defining markers are altered when treated with SF including HLA-DR. Our results reveal that ADSC maintain their immune-evasive properties even when exposed to SF and would not be rejected when injected into the articular microenvironment. We demonstrate as well that rheumatoid synovial fluids do not inhibit ADSC proliferation with 2 out of 8 even inducing proliferation. Maintaining ADSC proliferative properties is essential in order to maximize their effect in the local joint environment.

It has been previously demonstrated that the therapeutic ability of mesenchymal stem cells varies depending on the local environment they encounter (34). Rheumatoid synovial fluids reportedly contain high levels of pro-inflammatory cytokines (26) which suggests their potential role in ADSC immunomodulation. Our findings on the induction of immunomodulatory factors by inflammatory RASF lead to believe that RASF could indeed push ADSC potential forward. More specifically, IDO, PGE2, and IL-6 all play key roles in ADSC effects on the proliferation, phenotype and function of T lymphocytes and macrophages as blocking either one of these molecules mitigated ADSC-mediated immunomodulation (12, 15). Furthermore, ICAM-1, V-CAM1, and PD-L1, which were increased as well, are all involved in mediating the anchoring of T cells to ADSC and their subsequent functional inhibition whereas TSG6 is more implicated in skewing macrophages toward an anti-inflammatory phenotype (16, 35). These results are in accordance with a study by Leijs et al. that reported higher mRNA expression of IDO in MSC conditioned with RASF compared to control (36). Overall, RA1 which stood out as the most inflammatory appears as the prototypical SF capable of enhancing ADSC properties. This was further confirmed by the strong relationship found between SF content of TNF and IL-6 and the induction of ADSC gene expression. High concentrations of TNF and IL-6 could thus be a basis for prediction of ADSC efficiency within the synovial joint. However, specific characteristics of SF that could predict their effect on ADSC still need to be more precisely defined.

On the other hand, it is important to highlight that only one rheumatoid synovial fluid RA8 did not contain high levels of pro-inflammatory molecules and that this particular RASF inhibited the expression of COX2, IDO, and TSG6. Insufficient concentrations of pro-inflammatory cytokines could thus abolish the immunomodulatory effects of ADSC.

One of the pro-inflammatory cytokines known to elicit the immunosuppressive function of ADSC and MSC from different sources is TNF (37, 38). It has indeed been shown that IFN-γ along with TNF could regulate MSC efficiency although this is limited to the concentrations used and duration of exposure (38). Having not detected IFN-γ in RASF, we evaluated whether or not TNF alone or in combination with IL-6 could dictate the immunomodulatory potential of ADSC. Here, we show that the presence of TNF and IL-6 in SF is essential to enhance ADSC immunoregulatory response as shown by either neutralizing TNF and IL-6 in pro-inflammatory RASF or adding recombinant IL-6 and TNF to the lesser inflammatory RASF in order to mimic a pro-inflammatory environment. Although TNF was shown to be the most critical, IL-6 seems to play a role as well in enhancing ADSC functionality but only when in combination with TNF. Our data suggest that although IL-6 and TNF play an essential role in the enhancing effect of RA1 on ADSC gene expression, other pro-inflammatory cytokines such as IL-1β could be contributing to its effect as well.

Moreover, we demonstrate that the enhancement of ADSC potential signals though the NF-κB pathway as shown by the inhibition of IκB expression in ADSC after stimulation with pro-inflammatory RASF. Our finding was confirmed by the selective inhibition NF-κB activation in ADSC. These results are consistent with studies done by Dorronsoro et al. which reveals the importance of TNF-mediated activation of NF-κB for priming the immunosuppressive function in MSC (8) and by Luz-Crawford et al. that demonstrated that MSC deficient for peroxisome proliferator-activated receptor which is known to inhibit NF-κB signaling pathways have enhanced immunosuppressive properties (39). Our finding thus reveals the importance of the TNF/NF-κB axis in priming ADSC.

IDO and TSG6 levels were not affected by the inhibition of NF-kB. The effect of TNF is thus not limited to NF-κB but crosstalks with other pathways as well. IDO secretion has been reported to be also dependent on the JAK/STAT signaling pathway (40). Regarding TSG6, a study by Wang et al. showed regulation by IDO through its metabolite kynurenic acid which activates the aryl hydrocarbon receptor (AhR) that binds to the promoter of TSG6 and enhances its expression (41).

**Figure 7:**
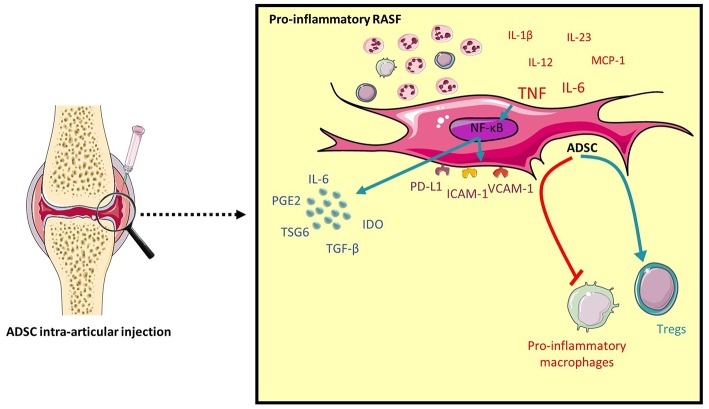
Pro-inflammatory RASF induce ADSC immunomodulatory potential. An intra-articular delivery of ADSC to RA patients would enhance their clinical benefits. High levels of pro-inflammatory cytokines, specifically TNF activates the NF-κB pathway in ADSC and induces the expression of COX2, IDO, IL-6, TSG6, ICAM-1, VCAM-1, and PD-L1. This will functionally enhance ADSC's ability to inhibit pro-inflammatory macrophages and induce Tregs.

ADSC have been shown to modulate T cell responses by generating regulatory T cells and inhibiting Th1 and Th17 cells (19, 42). In line with our results regarding a differential regulation of ADSC gene expression by SF, here we demonstrate that conditioning ADSC with pro-inflammatory RASF functionally enhances their ability to induce regulatory T cells compared to control SF due to the presence of TNF in RASF. In contrast, ADSC conditioned with lesser inflammatory RASF are less efficient in inducing Tregs. Similarly to their effects on T cells, priming ADSC with pro-inflammatory RASF functionally results in a more potent inhibition of the pro-inflammatory markers CD40 and CD80 on activated macrophages with no effect on alternative M2 markers. This could be explained by the relatively short time period of ADSC:macrophages co-culture to induce their expression. Altogether, our finding shows that ADSC exposed to a pro-inflammatory RASF exhibits a much stronger immunoregulatory response (Figure 7).

Overall, this study is the first to show that the proinflammatory environment of rheumatoid synovial fluids plays an essential role in orchestrating the immunomodulatory potential of ADSC. This opens the door for the consideration of the physiological levels of TNF and, for the first time, IL-6 when studying ADSC immunomodulatory plasticity in a clinical setting. Quantifying TNF and IL-6 in the articular joint prior to ADSC administration could indeed help predict their therapeutic outcome.

## Conclusion

In conclusion, our results suggest that the use of ADSC as a therapeutic strategy should take into account the influence of the local joint environment on their potential. Licensing of ADSC by an intra-articular delivery to inflamed joints would maximize their clinical benefits in the treatment of RA. However, a better understanding of mechanisms that regulate the immunomodulatory properties of mesenchymal stem cells remains of pivotal importance for ameliorating MSC-based therapies.

## Data Availability

The datasets generated for this study are available on request to the corresponding author.

## Ethics Statement

The use of ADSC in this study was approved by the Université Saint-Joseph ethics committee (CEHDE 733). The SF study protocol was approved by the CHU Toulouse ethics committee (BioTOUL DC 2016-2804).

## Author Contributions

SS designed the study, performed experimental work, analyzed and interpreted the data, and wrote the manuscript. NA and J-LD designed the study, interpreted the data, and critically revised the manuscript. EC, ArC, and AlC provided SF samples. OE and KD performed experimental work. BR, YD, and VT-S interpreted the data. All authors read and approved the final manuscript. NA and J-LD contributed equally to this work and both head their corresponding labs.

### Conflict of Interest Statement

The authors declare that the research was conducted in the absence of any commercial or financial relationships that could be construed as a potential conflict of interest.

## Abbreviations

ADSC: Adipose-derived mesenchymal stem cells
BM-MSC: bone marrow-derived stem cells
CCL,CXCL: Chemokine ligand
CD: cluster of differentiation
CTL: control
DAS 28-CRP: disease activity score 28 using C-reactive protein
Cyclooxygenase-2: COX2
DMEM/F12: Dulbecco's modified eagle medium/nutrient mixture F-12
ELISA: Enzyme-linked immunosorbent assay
EDTA: ethylenediaminetetraacetic acid
FBS: fetal bovine serum
Foxp3: forkhead box p3
HLA-DR: human leukocyte antigen-DR isotype
MCP-1: monocyte chemoattractant protein 1
MSC: mesenchymal stem cells
NK cells: natural killer cells
ICAM-1: intercellular adhesion molecule 1
IDO: indoleamine-1,2-dioxygenase
IFN-γ: interferon gamma
IL: interleukin
IL-1ra: interleukin-1 receptor antagonist
LPS: lipopolysaccharide
PBMC: peripheral blood mononuclear cells
PBS: phosphate buffered saline
PCR: polymerase chain reaction
PD-L1: programmed death-ligand 1
PGE2: prostaglandin E2
PMA: phorbol myristate acetate
PS: penicillin-streptomycin
RA: rheumatoid arthritis
SpA: Spondyloarthritis
RPS9: ribosomal protein S9
STC1: stanniocalcin-1
SVF: stromal-vascular fraction
TGF-β: transforming growth factor beta
TIMP3: tissue inhibitor of metalloproteinases 3
TNF: tumor necrosis factor
TSG6: tumor-necrosis factor stimulated gene 6
Th: T helper
Tregs: regulatory T cells
VCAM-1: vascular cell adhesion molecule 1.
