# Corrigendum: Rheumatoid Synovial Fluids Regulate the Immunomodulatory Potential of Adipose-Derived Mesenchymal Stem Cells Through a TNF/NF-κB-Dependent Mechanism

**DOI:** 10.3389/fimmu.2019.01961

**Published:** 2019-08-20

**Authors:** Souraya Sayegh, Oula El Atat, Katy Diallo, Benjamin Rauwel, Yannick Degboé, Etienne Cavaignac, Arnaud Constantin, Alain Cantagrel, Viviane Trak-Smayra, Nada Alaaeddine, Jean-Luc Davignon

**Affiliations:** ^1^Centre de Physiopathologie de Toulouse Purpan, INSERM UMR 1043, Toulouse, France; ^2^Université Paul Sabatier Toulouse III, Toulouse, France; ^3^Faculté de Médecine, Université Saint-Joseph, Beirut, Lebanon; ^4^Centre de Rhumatologie, CHU de Toulouse, Toulouse, France; ^5^Centre de Chirurgie Orthopédique et Traumatologique, CHU de Toulouse, Toulouse, France; ^6^Faculty of Medical Sciences, Neuroscience Research Center, Lebanese University, Beirut, Lebanon

**Keywords:** mesenchymal stem cells, rheumatoid arthritis, synovial fluid, immunomodulation, TNF, NF-κB, Tregs, macrophages

In the original article we neglected to indicate that both Dr. Nada Alaaeddine and Dr. Jean Luc Davignon contributed equally to this work.

A correction has also been made to the ***Author Contribution***statement, which appears below:

“SS designed the study, performed experimental work, analyzed and interpreted the data, and wrote the manuscript. NA and J-LD designed the study, interpreted the data, and critically revised the manuscript. EC, ArC, and AlC provided SF samples. OE and KD performed experimental work. BR, YD, and VT-S interpreted the data. All authors read and approved the final manuscript. NA and J-LD contributed equally to this work and both head their corresponding labs.”

The authors apologize for this error and state that this does not change the scientific conclusions of the article in any way. The original article has been updated.

