# Supplementary material for: Rheumatoid Synovial Fluids Regulate the Immunomodulatory Potential of Adipose-Derived Mesenchymal Stem Cells Through a TNF/NF-κB-Dependent Mechanism
Source: Front Immunol. 2019 Jun 28;10:1482. doi: 10.3389/fimmu.2019.01482 (PMC6611153; doi:10.3389/fimmu.2019.01482)
Supplement: Supplementary file 1 [file Data_Sheet_1.PDF]

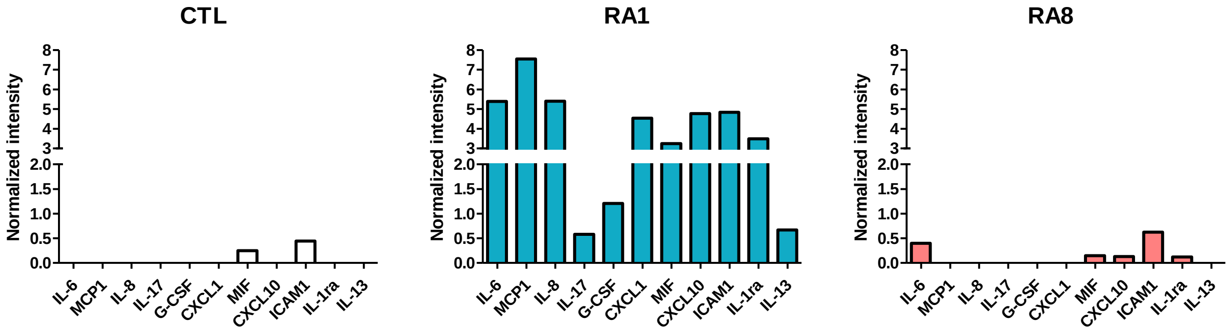


**Figure S1. Evaluation of synovial fluids inflammatory status.** Graphical representation of Figure 1A. SF were represented depending on their inflammatory status (blue being very pro-inflammatory, red slightly inflammatory). Pro-inflammatory mediators IL-6, MCP1, IL-8, IL-17, G-CSF, MIF, CXCL10, ICAM-1, IL-1RA, IL-13 were identified and semi-quantified using an antibody-based membrane array in CTL, RA1 and RA8 synovial fluids.

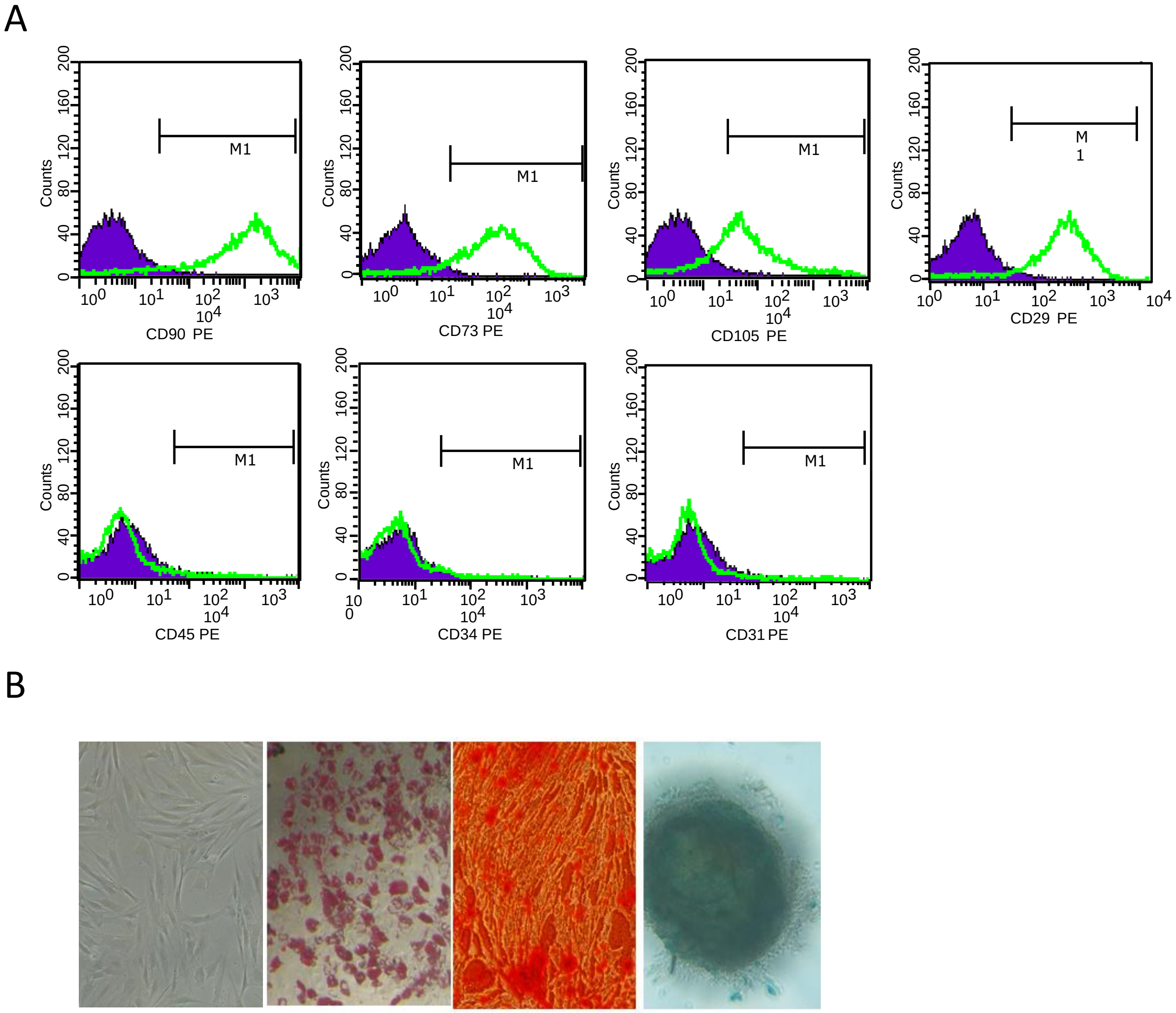


**Figure S2. Characterization of ADSC phenotype and differentiation potential. (A)** ADSC were stained with indicated antibodies and analyzed by flow cytometry. Histograms are representative of 5 independent experiments. Green lines represent stained cells and purple filled histograms their isotype-matched control. **(B)** ADSC were cultured in the presence of adipogenic, osteogenic or chondrogenic media for 21 days and then stained with either Oil Red O (middle left panel), Alizarin Red (middle right panel) and Alcian Blue (right panel) respectively. ADSC cultured alone served as a negative control (left panel) (magnification x10)

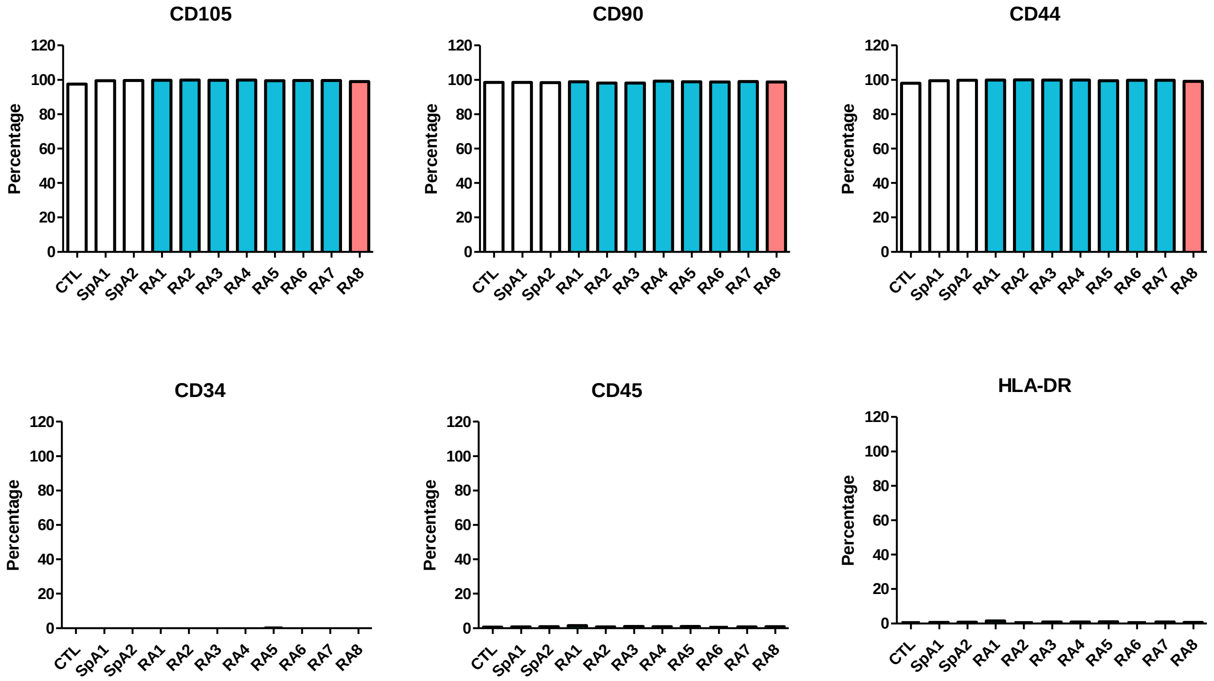


**Figure S3. The phenotype of ADSC is not altered after SF treatment.** ADSC were cultured for 24 hours in the presence of SF from either RA, SpA or CTL patients. Cells were then harvested and stained with anti-CD105, anti-CD90, anti-CD44, anti-CD34, anti-CD45 and anti-HLA-DR for their flow cytometry immunophenotyping.

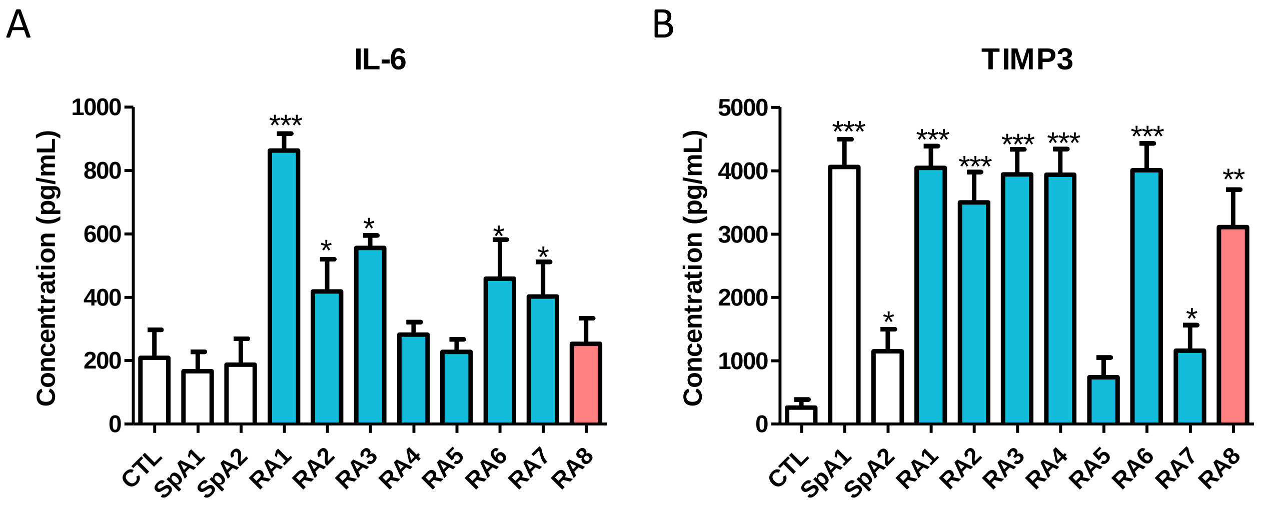


**Figure S4. Differential effect of synovial fluids on ADSC secretion of IL-6 and TIMP3.** ADSC were cultured for 24 hours in the presence of SF from either RA, SpA or CTL patients. **(A)** IL-6 and **(B)** TIMP3 were quantified in ADSC supernatants by ELISA. Results are represented as mean ± SEM of 5 independent experiments. *: $p<0.05$; **: $p<0.01$; ***: $p<0.001$.

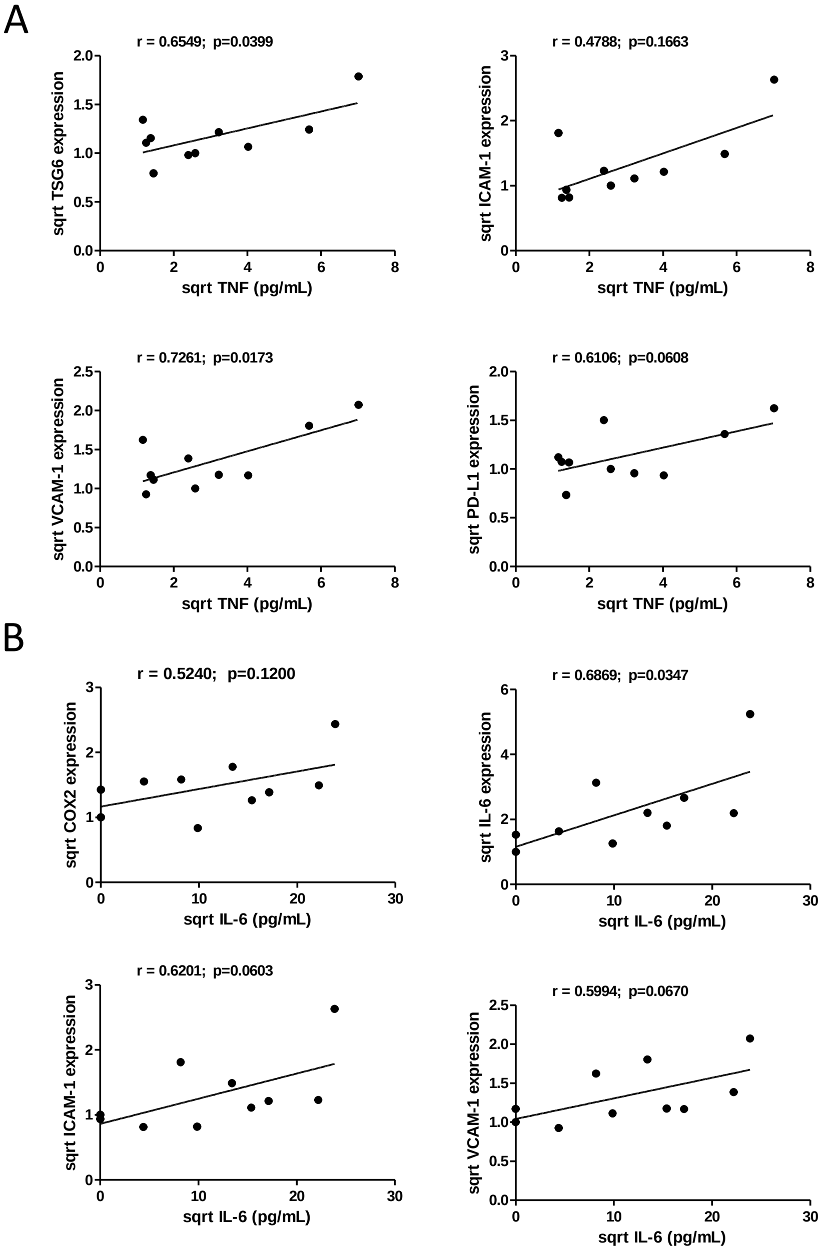


**Figure S5. Correlation between TNF and IL-6 SF concentrations and ADSC gene expression. (A)** Correlation (Pearson) between TNF concentrations and TSG6, ICAM-1, VCAM-1 and PD-L1 ADSC gene expression. **(B)** Correlation (Pearson) between IL-6 concentrations and COX-2, IL-6, ICAM-1 and VCAM-1 ADSC gene expression.

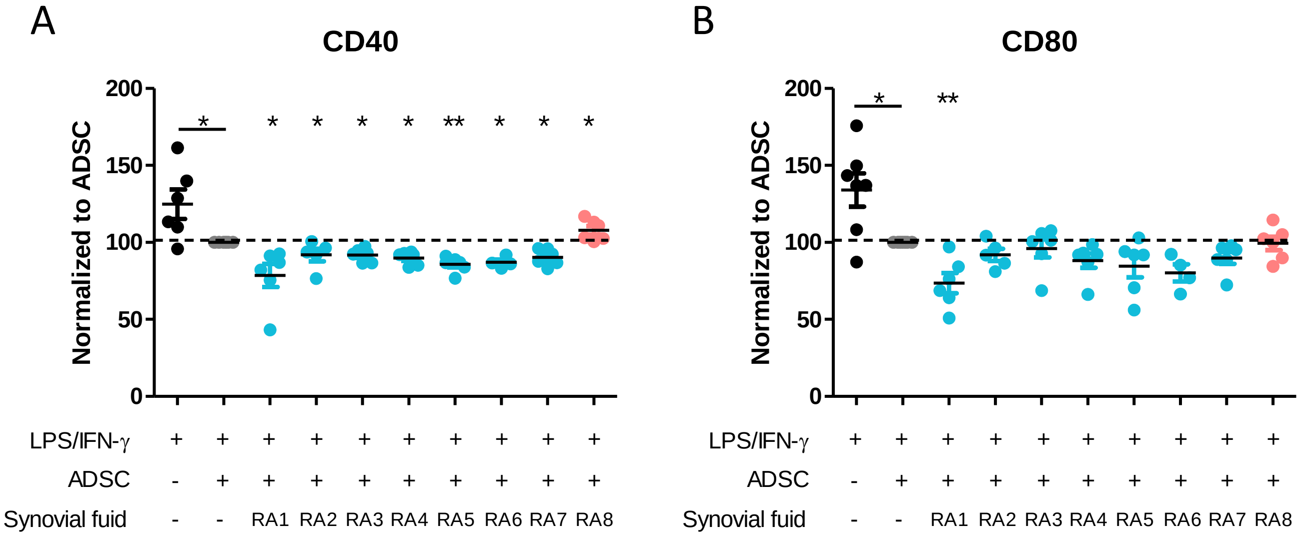


**Figure S6. Conditioning ADSC with pro-inflammatory RASF enhances their ability to inhibit CD40 and CD80 expression in macrophages.** ADSC were plated in 48-well plates and stimulated for 24 hours with RA1 to RA8. The following day, activated macrophages from healthy donors were added to ADSC at a ratio of 1:5 for 24 hours. Cells were then harvested and stained with anti-CD40 **(A)** and anti-CD80 **(B)** for flow cytometry detection of pro-inflammatory markers in macrophages. Results are represented as mean ± SEM of 4-7 independent experiments. Activated macrophages cultured in the presence of ADSC conditioned with different RASF were compared to those cultured in the presence of ADSC control. *: $p<0.05$; **: $p<0.01$.

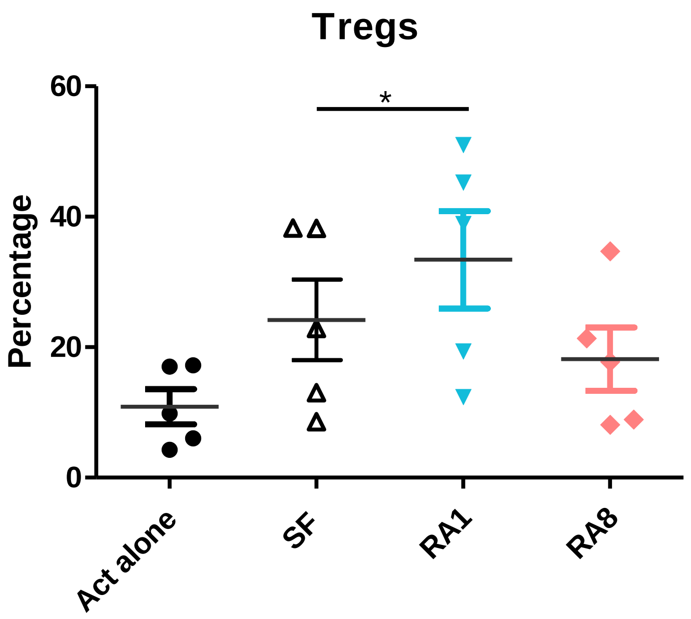


**Figure S7. Conditioning ADSC with pro-inflammatory RASF enhances their ability to induce Tregs in PBMC.** ADSC were plated in 96-well plates and stimulated for 24 hours with SF control, RA1 or RA8. The following day, ADSC were washed and PBMC cells from healthy donors were added to ADSC at a ratio of 1:5 and activated with beads coated with anti-CD3/CD28 for 72 hours. Cells were harvested and stained with anti-CD4, anti-CD25 and anti-Foxp3 for flow cytometry detection of Tregs. Results are represented as mean ± SEM of 5 independent experiments. *: $p<0.05$.

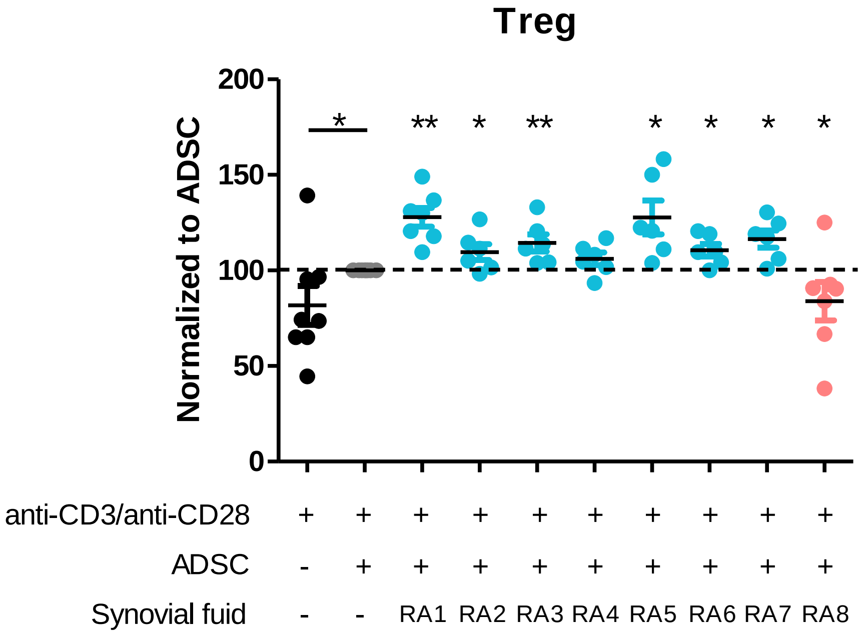


**Figure S8. Conditioning ADSC with pro-inflammatory RASF enhances their ability induce Tregs.** ADSC were plated in 96-well plates and stimulated for 24 hours with RA1 to RA8. The following day, ADSC were washed and T cells from healthy donors were added to ADSC at a ratio of 1:5 and activated with beads coated with anti-CD3/CD28 for 72 hours. Cells were then harvested and stained with anti-CD4, anti-CD25 and anti-Foxp3 for flow cytometry detection of Tregs. T cells activated in the presence of ADSC conditioned with different RASF were compared to those activated in the presence of ADSC control. Results are represented as mean ± SEM of 6-7 independent experiments. *: $p<0.05$; **: $p<0.01$.
