# Supplementary material for: Rheumatoid Synovial Fluids Regulate the Immunomodulatory Potential of Adipose-Derived Mesenchymal Stem Cells Through a TNF/NF-κB-Dependent Mechanism
Source: Front Immunol. 2019 Jun 28;10:1482. doi: 10.3389/fimmu.2019.01482 (PMC6611153; doi:10.3389/fimmu.2019.01482)
Supplement: Supplementary file 2 [file Table_1.DOCX]

**Table S1. List of primer sequences**

| **Gene** | **Forward** | **Reverse** |
| --- | --- | --- |
| RPS9 | GATTACATCCTGGGCCTGAA | ATGAAGGACGGGATGTTCAC |
| COX-2 | TGACCAGAGCAGGCAGATGAA | CCACAGCATCGATGTCACCATAG |
| IDO | GCCCTTCAAGTGTTTCACCAA | CCAGCCAGACAAATATATGCGA |
| IL-6 | CCACACAGACAGCCACTCAC | CCAGATTGGAAGCATCCATC |
| TSG-6 | AGGCGGTGTGTGAATTTGAAG | GGCTGCCTCTAGCTGCTTGT |
| ICAM-1 | CCTTCCTCACCGTGTACTGG | AGCGTAGGGTAAGGTTCTTGC |
| VCAM-1 | GGCGCCTATACCATCCGAAA | AGAGCACGAGAAGCTCAGGAGAA |
| PD-L1 | GGCATCCAAGATACAAACTCAA | CAGAAGTTC CAATGCTGGATTA |
| TGF-BETA | CGCGTGCTAATGGTGGAAA | TGTGTGTACTCTGCTTGAACTTGTCA |
| TIMP3 | CAGGACGCCTTCTGCAACTC | AGCTTCTTCCCCACCACCTT |
| STC1 | GTGGTTCGTTGCCTCAACAGT | GTGTCACAGGTGGAGTTTTCCA |
